# Supplementary figures and images for: Selection of appropriate reference genes for quantitative real-time reverse transcription PCR in Betula platyphylla under salt and osmotic stress conditions
Source: PLoS One. 2019 Dec 3;14(12):e0225926. doi: 10.1371/journal.pone.0225926 (PMC6890252; doi:10.1371/journal.pone.0225926)

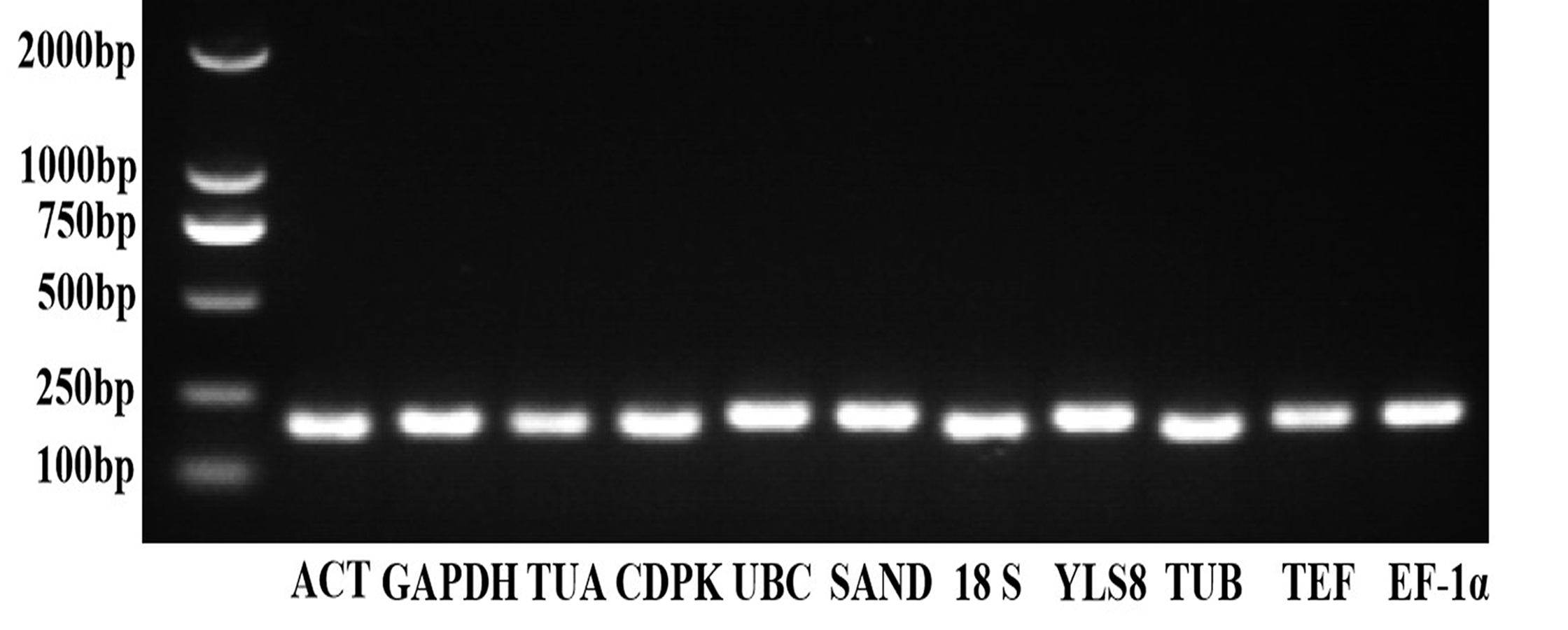

Supplement: S1 Fig — (TIF) [file pone.0225926.s001.tif]

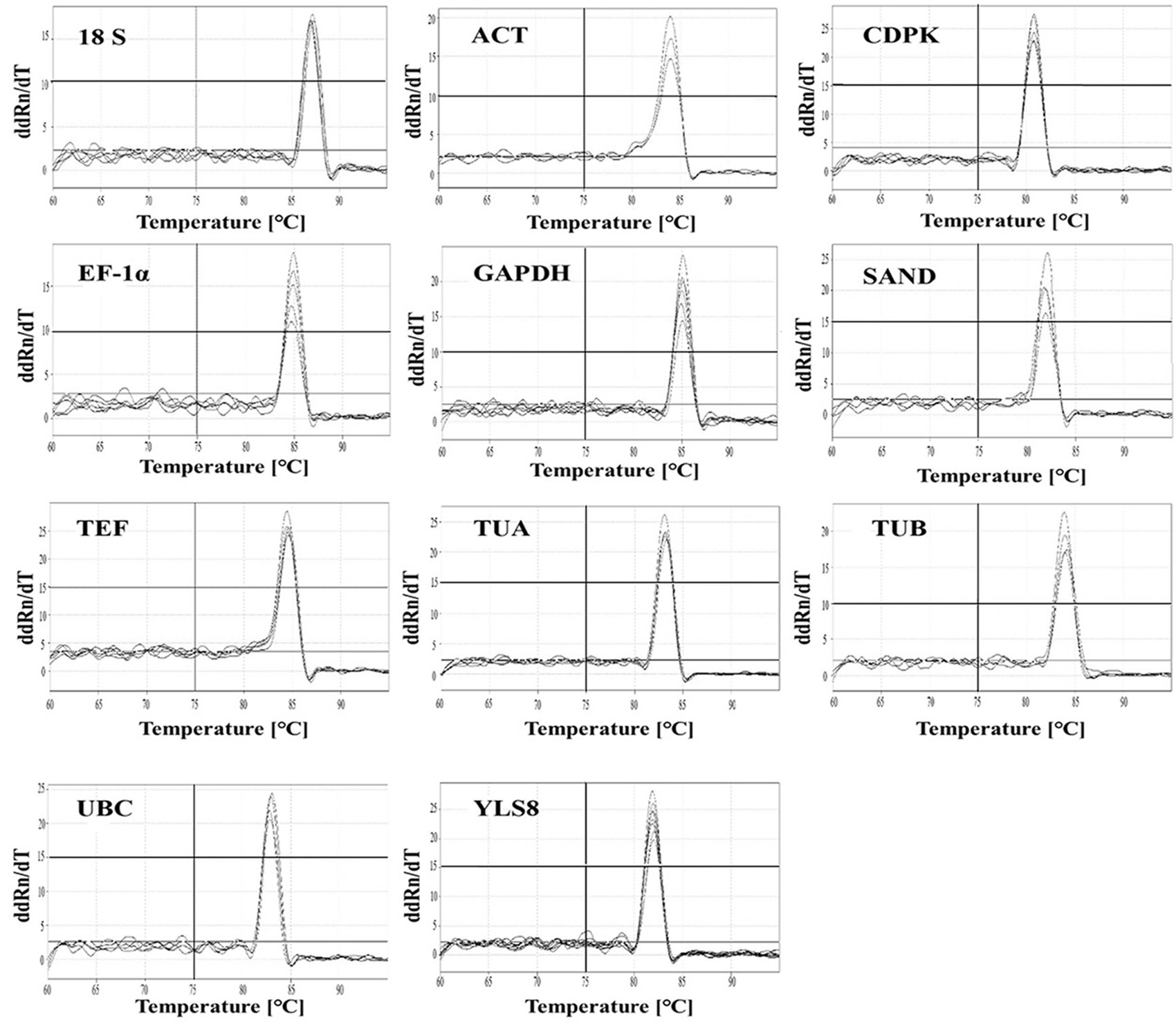

Supplement: S2 Fig — (TIF) [file pone.0225926.s002.tif]
