# Supplementary material for: Selection of appropriate reference genes for quantitative real-time reverse transcription PCR in Betula platyphylla under salt and osmotic stress conditions
Source: PLoS One. 2019 Dec 3;14(12):e0225926. doi: 10.1371/journal.pone.0225926 (PMC6890252; doi:10.1371/journal.pone.0225926)
Supplement: S1 Table — GN: Ranking of candidate reference genes calculated by geNorm. NF: Ranking of candidate reference genes calculated by NormFinder. BK: Ranking of candidate reference genes calculated by Bestkeeper (DOCX) [file pone.0225926.s003.docx]

**S1 Table :** Comprehensive rankings of the stability of the reference genes by three algorithms.

| **Gene** | **Different tissues** | | |  | **Normal conditions** | | |  | **Salt stress** | | |  | **Osmotic stress** | | |  | **Total** | | |
| --- | --- | --- | --- | --- | --- | --- | --- | --- | --- | --- | --- | --- | --- | --- | --- | --- | --- | --- | --- |
|  | **GN** | **NF** | **BK** |  | **GN** | **NF** | **BK** |  | **GN** | **NF** | **BK** |  | **GN** | **NF** | **BK** |  | **GN** | **NF** | **BK** |
| *ACT* | 4 | 4 | 5 |  | 1 | 4 | 2 |  | 1 | 2 | 3 |  | 1 | 4 | 2 |  | 1 | 2 | 1 |
| *TUA* | 2 | 8 | 2 |  | 1 | 6 | 4 |  | 3 | 5 | 2 |  | 3 | 7 | 3 |  | 1 | 3 | 2 |
| *TUB* | 1 | 5 | 3 |  | 2 | 1 | 5 |  | 2 | 3 | 4 |  | 1 | 2 | 1 |  | 2 | 1 | 3 |
| *YLS8* | 7 | 6 | 9 |  | 6 | 5 | 8 |  | 8 | 9 | 10 |  | 7 | 5 | 5 |  | 3 | 4 | 6 |
| *SAND* | 6 | 3 | 7 |  | 5 | 3 | 9 |  | 6 | 7 | 7 |  | 5 | 6 | 7 |  | 4 | 6 | 7 |
| *UBC* | 8 | 9 | 8 |  | 9 | 10 | 10 |  | 5 | 6 | 6 |  | 8 | 9 | 9 |  | 5 | 5 | 9 |
| *TEF* | 3 | 7 | 1 |  | 8 | 9 | 6 |  | 4 | 1 | 1 |  | 2 | 1 | 4 |  | 6 | 7 | 5 |
| *EF1α* | 1 | 2 | 4 |  | 3 | 2 | 7 |  | 1 | 4 | 5 |  | 4 | 3 | 6 |  | 7 | 8 | 8 |
| *CDPK* | 9 | 10 | 10 |  | 4 | 8 | 1 |  | 7 | 10 | 9 |  | 6 | 8 | 8 |  | 8 | 9 | 10 |
| 18S *rRNA* | 5 | 1 | 6 |  | 7 | 7 | 3 |  | 9 | 8 | 8 |  | 9 | 10 | 10 |  | 9 | 10 | 4 |
| *GAPDH* | 10 | 11 | - |  | 10 | 11 | - |  | 10 | 11 | - |  | 10 | 11 | - |  | 10 | 11 | - |

GN: Ranking of candidate reference genes calculated by geNorm

NF: Ranking of candidate reference genes calculated by NormFinder

BK: Ranking of candidate reference genes calculated by Bestkeeper
