# Supplementary material for: Selection of appropriate reference genes for quantitative real-time reverse transcription PCR in Betula platyphylla under salt and osmotic stress conditions
Source: PLoS One. 2019 Dec 3;14(12):e0225926. doi: 10.1371/journal.pone.0225926 (PMC6890252; doi:10.1371/journal.pone.0225926)
Supplement: Attachment — Submitted filename: Cover letter and Response to reviewers.docx [file pone.0225926.s004.docx]

Cover letter

Dear editor,

We are very grateful to your and the reviewers’ valuable suggestions. Based on these suggestions, we have made careful modification on the original manuscript. We also responded point by point to the reviewer’s comments as listed below. Hope these will make it more acceptable for publication. If there are further issues to be clarified, please contact us without hesitation. Thank you.

With best wishes,

Xiaoyu Ji

College of Forestry, Shenyang Agricultural University, Shenyang, 110866, China.

E-mail address: jixiaoyu@syau.edu.cn

Response to Reviewer Comments

**Reviewer 1**

**Critical points:**

**1.** Multiple parameters need to consider before choosing an ideal reference gene for qPCR analysis. Among them, one of the very important criteria is qPCR efficiency, and it should be close to 2 (within 80–100%). The authors used E^-ΔCt^ formula for data analysis. I assume ‘E’ stands for qPCR efficiency. Therefore, it is necessary to measure the qPCR efficiency of each gene and mention in the manuscript. Apart from that, all other criteria are satisfactory: single pick dissociation curve, single band in agarose gel, and amplicon size.

**Response:** We have added PCR efficiency (E) in Table 1.(see line: 152-154, 233-234.)

**2.** Authors tried to summarize the analysis of three statistical algorithms (GeNorm, NormFinder, and BestKeeper) by calculating the ‘sum of rankings’ in table 4. Before that, it needs to establish the robustness of data by correlation analysis (R^2^). Example: correlation analysis between M Value vs Stability Value. After that, authors can perform a sum of rankings analysis using the best-correlated algorithms or all algorithms.

**Response:** We have established the robustness of data by correlation analysis (R^2^) between M Value vs Stability Value, M Value vs Stability Value. (see line: 176-177, 400-402.)

**3.** Statistical analysis is missing in figure 3 and 4. I would suggest performing a simple T-test.

**Response:** We have added it according to the suggestion. T-test was performed to analyze its significanced, and the results were shown in Figure 3 and Figure 4. (see line: 357-371.)

**4.** Sampling and calculation procedure is not clear. I assume authors harvested root, stem and leaf tissues from normal and stressed samples after the given time points. How many samples were used per experiments? Are they biological or technical replication? Also, maintain uniformity in the text; either use “Normal” or “Control”. In addition, authors should clarify how they calculate average for each groups (i.e., tissue, normal, salt and osmotic).

**Response:** We have explained it according to the suggestion. Biologically repeated sampling from 6 birch seedlings in three independent experiments. Three independent replications were performed for technical repetition in qRT-PCR. The mean value is arithmetic mean.(see line: 120, 154.)

**Minor points:**

**1.** Line 65: All are not woody plants. Example. *Musa paradisiaca*.

**Respon**se: We have deleted the relevant reference according to the suggestion. (see line: 87.)

**2.** Line 100: Mention cycle no in the Fig.A1 legend.

**Response:** We have added mentioned cycle in the Figure S1 according to the suggestion. (see line: 202.)

**3.** Line 113: Range of the mean Ct values (17.34 to 28.27) is not matching with Table 1 (17.39 to 29.80).

**Response:** We have corrected it according to the suggestion. The range of the mean Ct values is 17.39~29.80. (see line: 214.)

**4.** Line 138: To follow the result easily, mention the “four groups” here as well. Also, describe “total” sample.

**Response:** Four groups means ‘different tissues, normal, salt, and osmosis’. Total number of samples called ‘total sample’, and we have corrected it according to the suggestion. (see line: 245-246.)

**5.** Line 142-145: Some data points are missing in Figure 2. Ex: *EF-1α* in different tissues and *ACT* in salt stress.

**Response:** Missing data points (*EF-1α* in different tissues, *ACT* in salt stress, *ACT* in normal condition) have been completed. We have reconstructed the Figure 2 according to the suggestion.

**6.** Line 145: Use uniform terminology in the manuscript. Either “all samples” or “total samples”.
**Response:** We have corrected it according to the suggestion. Use uniform terminology ‘total sample’. (see line :253.)

**7.** Line 151: Authors should explain the calculation procedures in details in Figure 2 legend. How they calculated “the average expression stability (M)” values for normal and stressed samples? I assume the authors averaged the data from different time points as well as different tissues. Also, they should mention the replication no. I would recommend this suggestion for other figures and tables as well.

**Response:** We have added it according to the suggestion. Detailed calculation process has been demonstrated. (see line :238-242.)

**8.** Line 132 & 155: Numbers are same for both sections.

**Response:** We have corrected number according to the suggestion.(see line: 206, 235.)

**9.** Line 172: Are not “M values” stands for geNorm analysis? If so, correct Table 2 as it is showing values from NormFinder analysis.

**Response:** The Normfinder software processing results are SV values. We have corrected it according to the suggestion. (see line :274.)

**10.** Line 183: I think authors should explain why they choose SD value over the R-value for the ranking.

**Response:** More references were ranked with SD values, which was more convincing, however R values can also be used as ranking criteria. We have explained it in the revised manuscript. (see line :288-290.)

**11.** Line 186-187: “On the whole…. experimental conditions used”; Do you mean normal condition?

**Respons**e: Yes, it was normal condition. We have corrected it according to the suggestion. (see line :298.)

**12.** Line 188-189: “Under salt stress conditions…. used as reference genes”; In this situation authors can consider the R-value to choose best one.

**Respons**e: We have taken this method according to the suggestion. Compared R values in this case and find that the R values of *ACT* and *EF1α* were closer to 1, so we choosed them. (see line :300-301.)

**13.** Line 192: “Ultimately, ACT…. to the results”; Mention the sample name.

**Response:** We have corrected it according to the suggestion. (see line :304.)

**14.** Line 202: The authors validated well the best reference genes through expression analysis of GRAS TFs in salt-stressed samples. To do that, the authors cited multiple references that showed stressed-mediated expression of GRAS homologs. However, they did not cite any reference for tissue-specificity. I would recommend adding some references to show that the GRAS TFs can be expressed tissue-specific manner.
**Response:** We have cited relevant reference to illustrate the tissue specificity of GRAS TFs expression according to the suggestion. (see line :318-320.)

**15.** Line 255: I think it will be “A2-C2” instead of “B2-C2”.

**Response:** We have corrected it according to the suggestion. (see line :362.)

**16.** Line 293: Authors mentioned about cloning. However, no such section available in the materials and method.

**Response:** In the previous study, we have obtained the sequence of these 11 genes. We have mentioned it according to the suggestion. (see line :394.)

**17.** Line 303: Why not TUB for osmotic stress. It showed lowest sum of rankings.

**Response:** TUB ranks better than TEF. We have corrected it according to the suggestion. (see line :45, 405.)

**18.** Line 342-349: Mention RNA amount that were used for cDNA synthesis.

**Response:** We have added it in the material method according to the suggestion. (see line :130-131.)

**19.** Line 351-356: Mention Tm of the primers.

**Response:** We have added it in Table 1 according to the suggestion. (see line :233.)

**20.** Line 367-368: Mention cut-off/ threshold for the Ct values (if manually adjusted).

**Response:** The cycle threshold (Ct) values is the machine setting. We have explained it in the revised manuscript. (see line :159-160.)

**21.** Line 370: How many biological and/or technical replications were used for mean Ct value calculation?

**Response:** The mean Ct value was calculated using three biological replications, we have added it according to the suggestion. (see line :160.)

**Reviewer 2**

**General comments:**

**1.** The description of results and discussion are jittered and need to be rephrased to get a seamless flow.

**Response:** We have reworded inappropriate and redundant parts according to the suggestion.

**2.** Excessive references (65) that doesn’t add more value to the paper may be restricted to max.40. More of recent and references of woody species should be included eg.:
a. Wang JJ, Han S, Yin W, Xia X, Liu C. Comparison of Reliable Reference Genes Following Different Hormone Treatments by Various Algorithms for qRT-PCR Analysis of *Metasequoia*. International Journal of Molecular Sciences. 2018. 20(1):34.
b. Wei Y, Liu Q, Dong H, et al. Selection of Reference Genes for Real-Time Quantitative PCR in *Pinus massoniana* Post Nematode Inoculation. PLoS One. 2016;11(1):e0147224.
c. Wang J, Abbas M, Wen Y, Niu D, Wang L, Sun Y, et al. (2018) Selection and validation of reference genes for quantitative gene expression analyses in black locust (*Robinia pseudoacacia L.*) using real-time quantitative PCR. PLoS ONE 13(3): e0193076. https://doi.org/10.1371/journal.pone.0193076
d. Chen X, Mao Y, Huang S, et al. Selection of Suitable Reference Genes for Quantitative Real-time PCR in *Sapium sebiferum*. Front Plant Sci. 2017;8:637.

**Response:** We have read these four articles in detail and cited them, while ensuring that the references are not redundant, within 40 articles according to the suggestion.

**3.** The results, few sentences verbatim is repeated in the abstract, results and discussion – which might be rephrased.

**Response:** We have corrected them according to the suggestion, eg.: see line: 108.

**Major:**

**1.** Line 65 – Ref 29 is either misplaced/ missing

**Response:** We have deleted it according to the suggestion. (see line :87.)

**2.** Line 66 – Triticum is not a woody plant – and including this example doesn’t add any new information.

**Response:** We have deleted it according to the suggestion. (see line :89.)

**3.** In the - ‘Validation of reference genes’ results section paragraphs starting from line 220 to 235 is not clear and the reader gets lost. Consider rephrasing the paras.

**Response:** We have corrected it and deleted corresponding unclear segment according to the suggestion. (see line :334-336.)

**4.** Line 287 – Too many references may be avoided, to keep it concise. The given literature has been previously cited in the introduction section also. Eg.: Line 288 – reference 52 – the original paper has No PCR studies reported in this paper and hence irrelevant.

Redundant reference has been.

**Response:** We have deleted it according to the suggestion.(see line :388-389.)

**5.** Line 317 – wrong reference cited (44) instead it should be 62.

**Response:** We have corrected it according to the suggestion. (see line : 416.)

**6.** Line 321 – ref. Wan et al., 2010 – not in reference.

**Response:** We have cited this reference according to the suggestion. (see line : 419.)

**7.** Line 323 – wrong reference – referring to animal sciences paper not *Magnolia denudate.*

**Response:** We have deleted it according to the suggestion. (see line : 420.)

**8.** Line 334 – the source of birch seeds should be mentioned.

**Response:** We have mentioned birch seeds source in the revised manuscript. (see line : 113-114.)

**9.** Line 337 – the quantity (in ml or L) used each time may be mentioned. Whether tap water or distilled water was used?

**Response:** We have mentioned volume of distilled water according to the suggestion. (see line : 117-118.)

**10.** Line 424 – ref.10 – non relevant animal studies paper may be omitted.

**Response:** We have deleted it according to the suggestion.

**11.** Line 475 – ref.29 is NOT a ‘validation’ paper.

**Response:** We have deleted it according to the suggestion.

**12.** Line 536 – ref.52 - no RT-PCR studies reported in the paper, hence irrelevant.

**Response:** We have deleted it according to the suggestion.

**13.** Line 567 – ref.63 – irrelevant reference.

**Response:** We have deleted it according to the suggestion.

**Minor:**

**1.** Line 39 – maybe modified as “food spoilage detection” to be specific.

**Response:** We have corrected it according to the suggestion. (see line : 61.)

**2.** Line 40 – just TWO important references would suffice to support.

**Response:** We have deleted redundant references according to the suggestion. (see line : 62.)

**3.** Line 74 and 78 – not clear – consider rephrasing.

**Response:** We have rephrased it according to the suggestion.(see line : 94-98.)

**4.** Line 80 – a couple of recent literature would suffice.

**Response:** We have deleted redundant references according to the suggestion.(see line : 101.)

**5.** Line 86 – The sentence is repeated in the abstract verbatim, which may be avoided.

**Response:** We have rephrased repeated words according to the suggestion. (see line : 108.)

**6.** Line 92 – “reached” may be replaced with “fulfilled”

**Response:** We have corrected it according to the suggestion. (see line : 196.)

**7.** Line 94 – “repeats” may be replaced with “replications”

**Response:** repeats --> replications. We have corrected it according to the suggestion. (see line : 198.)

**8.** Line 96 – “subjects” may be replaced with “candidates”

**Response:** We have corrected it according to the suggestion. (see line : 199.)

**9.** Superfluous ‘the’ may be omitted – eg. Line 106 and few other paragraphs.

**Response:** We have corrected it according to the suggestion. (see line : 208, 209.)

**10.** Line 118 – The result is repeated elsewhere and also in the same para line 114.

**Response:** We have removed repeated results according to the suggestion. (see line : 218-219.)

**11.** Line 340 – ‘cryotheraphy’ may be replaced with ‘after freezing in liquid nitrogen’

**Response:** We have corrected it according to the suggestion. (see line : 121.)

**12.** Line 341 – ‘experiments’ may be replaced with ‘replications’

**Response:** experiments --> replications.We have corrected it according to the suggestion. (see line : 120.)

**13.** Line 344 – ‘detected’ may be replaced with ‘checked’

**Response:** We have corrected it according to the suggestion. (see line : 127.)

**14.** Line 350 – ‘synthetic’ may be replaced with ‘synthesized’

**Response:** We have corrected it according to the suggestion. (see line : 133.)

**15.** Line 353 – too many references, repeated.

**Response:** We have deleted redundant references according to the suggestion. (see line : 138.)

**16.** Line 365 – ‘experiments’ may be replaced with ‘replications’

**Response:** experiments --> replications. We have corrected it according to the suggestion. (see line : 154.)

**17.** Line 393 – the non-standard phrase ‘depended on the way of’ may be replaced with ‘as described in’

**Response:** We have corrected it according to the suggestion. (see line : 190.)
